# Impact of 1-Year Supplementation with High-Rich Docosahexaenoic Acid (DHA) on Clinical Variables and Inflammatory Biomarkers in Pediatric Cystic Fibrosis: A Randomized Double-Blind Controlled Trial

**DOI:** 10.3390/nu16070970

**Published:** 2024-03-27

**Authors:** Roser Ayats-Vidal, Montserrat Bosque-García, Begoña Cordobilla, Oscar Asensio-De la Cruz, Miguel García-González, Susana Loureda-Pérez, Elena Fernández-López, Eva Robert-Barriocanal, Andrea Valiente-Planas, Joan Carles Domingo

**Affiliations:** 1 Pediatric Allergies, Immunology and Pneumology Unit, Pediatric Medicine Service, Institut d’Investigació i Innovació Parc Taulí (I3PT-CERCA), Parc Taulí Hospital Universitari, Universitat Autònoma de Barcelona, Parc Taulí 1, E-08208 Sabadell, Spain; bosquegarciamontse@gmail.com (M.B.-G.); oasensio@tauli.cat (O.A.-D.l.C.); mgarciao@tauli.cat (M.G.-G.); sloureda@tauli.cat (S.L.-P.); 2 Department of Biochemistry and Molecular Biomedicine, Faculty of Biology, University of Barcelona, E-08028 Barcelona, Spain; bgcordobilla07@ub.edu; 3 Physical Medicine and Rehabilitation Service, Institut d’Investigació i Innovació Parc Taulí (I3PT-CERCA), Parc Taulí Hospital Universitari, Universitat Autònoma de Barcelona, Parc Taulí 1, E-08208 Sabadell, Spain; efernandezl@tauli.cat (E.F.-L.); erobert@tauli.cat (E.R.-B.); avaliente@tauli.cat (A.V.-P.)

**Keywords:** cystic fibrosis, omega-3 fatty acids, docosahexaenoic acid, pulmonary function, inflammatory biomarkers, randomized controlled trial

## Abstract

A randomized, double-blind, and placebo-controlled study was conducted to assess the effect of dietary supplementation with high-rich docosahexaenoic acid (DHA) (Tridocosahexanoin-AOX^®^ 70%) at 50 mg/kg/day in pediatric patients with cystic fibrosis (CF) as compared with placebo. The duration of supplementation was 12 months. A total of 22 patients were included, with 11 in the DHA group and 11 in the placebo group. The mean age was 11.7 years. The outcome variables were pulmonary function, exacerbations, sputum cellularity, inflammatory biomarkers in sputum and peripheral blood, and anthropometric variables. In the DHA group, there was a significant increase in FVC (*p* = 0.004) and FVE_1_ expressed in liters (*p* = 0.044) as compared with placebo, and a lower median number of exacerbations (1 vs. 2). Differences in sputum cellularity (predominantly neutrophilic), neutrophilic elastase, and sputum and serum concentrations of resolvin D1 (RvD1), interleukin (IL)-8 (IL-8), and tumor necrosis factor alpha (TNF-α) between the study groups were not found. Significant increases in weight and height were also observed among DHA-supplemented patients. The administration of the study product was safe and well tolerated. In summary, the use of a highly concentrated DHA supplement for 1 year as compared with placebo improved pulmonary function and reduced exacerbations in pediatric CF.

## 1. Introduction

Adequate nutrition, including the intake of essential fatty acids (EFAs), particularly omega-3 and omega-6 polyunsaturated fatty acids (*n*-3 and *n*-6 PUFAs), is essential in patients with cystic fibrosis (CF) because of the high incidence of EFA deficiency and the role played by this deficiency in fat malabsorption and symptoms and the progression of pulmonary disease [1,2]. Although the prognosis of CF has remarkably improved with the introduction of modulators to target the specific mutations of the cystic fibrosis transmembrane conductance regulator (*CFTR*) gene [3], there has been limited progress in defining the underlying mechanisms involved in altered EFA metabolic pathways [4,5] and the anti-inflammatory effect of the dietary modulation of EFA deficiency [6,7].

Abnormal EFA profiles in the lipid membranes of CF patients are typically characterized by decreased levels of docosahexaenoic acid (DHA) and linoleic acid (LA), which is the precursor of arachidonic acid (AA), and an increased level of the AA/DHA ratio [8]. The intrinsic imbalance in the ratio of fatty acids with an excess of AA and a deficiency of DHA is critical in regulating the risk of inflammatory changes in the pathological pathways of a number of organ systems, including the lungs [1,7]. The release of AA from membranes via phospholipase A2 is the rate-limiting step for eicosanoid synthesis and is increased in CF, which contributes to the observed inflammation [8,9]. A potential deficiency of DHA may lead to decreased levels of specialized pro-resolving mediators, which serve as a novel class of immunoresolvents that promote the resolution of non-infectious inflammation and tissue injury [10]. Abnormalities in EFAs, which are involved as drivers of the pathophysiology of inflammation in CF, have provided the rationale for taking advantage of the anti-inflammatory effects associated with dietary supplementation with omega-3 fatty acids, such as those derived from fish oils [11,12,13]. Although omega-3 supplementation may provide some benefits for people with CF with relatively few adverse effects, evidence based on data of systematic reviews seems to be insufficient to recommend routine use or to draw firm conclusions [14,15].

A randomized controlled study aimed to characterize the EFA profile in the erythrocyte membrane of pediatric CF patients after a 1-year administration of a high-rich DHA supplement (Tridocosahexanoin-AOX^®^ 70%) at 50 mg/kg/day; the data revealed improvements in DHA and eicosapentaenoic acid (EPA) levels and decreases in *n*-6 PUFAs, primarily due to a decrease in AA [16]. Increases in DHA and EPA levels and decreases in AA accounted for lower AA/DHA and AA/EPA ratios, lower elongase-5 activity, and a higher anti-inflammatory fatty acid index (AIFAI) at the end of supplementation [16]. The aim of the present study was to assess the effect of this high-rich DHA triglyceride product on clinical variables, including pulmonary function, rate of exacerbations, and anthropometric parameters, as well as sputum and serum inflammatory biomarkers when administered for 1 year to children with CF. The assessment of these meaningful clinical outcomes will contribute to further defining the role of DHA supplementation as a potential anti-inflammatory therapy in pediatric CF. 

## 2. Materials and Methods

### 2.1. Study Design and Participants

This was a single-center randomized, double-blind, and controlled study carried out in daily practice conditions at the CF Pediatric Unit of an acute-care teaching hospital in Sabadell, Barcelona, Spain. The objective of the study was to assess changes in anthropometric, respiratory, and inflammatory variables in pediatric patients with CF treated with a high-rich DHA supplement for 12 months in comparison with placebo-treated patients. The results of an extensive biochemical profile of EFA families in the erythrocyte membrane of this population, which was also an objective of the same trial, have been previously reported [16]. Briefly, CF patients controlled at the hospital were eligible if they were 6–18 years old and had a forced expiratory volume in one second (FEV_1_) > 40%. Main exclusion criteria were SpO2 < 92%, use of domiciliary oxygen, current treatment with *CFTR* modulators, treatment with systemic steroids and/or NSAIDs in the previous 2–4 weeks, and current use of DHA supplementation.

The study protocol was approved by the institutional review board (code PED-DHA-2017, approval 18 November 2017). The study was registered on ClinicalTrials.gov (NCT04987567). Participants aged 18 years provided written informed consent. An informed assent statement was signed by participants aged between 12 and 17 years of age. Parents or legal guardians of participants aged ≤17 years were required to sign the informed consent document.

### 2.2. Randomization and Intervention

Participants were randomly assigned in a 1:1 ratio using a permuted block design, with each block containing either 4 or 6 subjects. They were allocated to either the experimental group (DHA supplementation) or the control group (placebo supplementation). Randomization was carried out by an independent investigator using a table of random numbers.

The active product consisted of high-rich DHA triglyceride (Tridocosahexanoin-AOX^®^ 70%) formulated in gelatin capsules (Brudy NEN, BrudyLab, S.L., Barcelona, Spain) that includes 400 mg of *n*-3 PUFAs (350 mg of pure DHA and 42.5 mg of EPA). This highly concentrated DHA triglyceride with a high antioxidant activity was patented for preventing cellular oxidative damage [17,18] and registered in the Spanish Agency for Consumer Affairs Food Safety and Nutrition (AESAN) as a food supplement. A daily dose of 50 mg/kg was selected because of bioavailability and safety data [19,20] and its use in a previous randomized controlled trial carried out in CF Units in Spain [21]. The corresponding daily doses according to the patient’s weight are shown in Table 1. Treatment in controls followed the same schedule and consisted of capsules of olive oil of the same organoleptic properties. The duration of dietary supplementation was 12 months.

Usual CF medications were allowed, and all patients continued with their prescribed treatments.

### 2.3. Study Procedures

Patients attended all study visits at the investigational center. The study included a screening visit (visit 1), a baseline visit (visit 2), and four visits at 3, 6, 9, and 12 months (visits 3, 4, 5, and 6), respectively. The screening visit took place within ±7 days of the baseline visit, in which the inclusion criteria were checked, the written informed consent was obtained, and randomization was performed. Data concerning medical history and the results of a physical examination were recorded.

At the baseline visit (visit 2), the study product was provided, and the following procedures were performed: clinical evaluation (anthropometric variables and review of exacerbation episodes within the last year), induced sputum test, spirometry, and a fasting blood sample drawn for laboratory analysis. At visit 3, the study product was provided and returned capsules were counted, a spirometry was performed, patients underwent a clinical evaluation, respiratory exacerbations were reviewed, and adverse events were assessed. At visit 4, the same procedures as in the baseline visit were performed, including assessment of exacerbation episodes since the last visit and adverse events. At visit 5, the same procedures as those in visit 3 were performed. At visit 6 (end of study), induced sputum, spirometry, clinical assessment, review of respiratory exacerbations, assessment of adverse events, safety, and compliance with treatment were recorded. Capsules returned were counted and adherence was defined as consumption of at least 80% of capsules.

### 2.4. Study Variables

Respiratory exacerbations were defined as an increase in dyspnea unrelated to other causes associated or not with other symptoms (such as cough, fever, asthenia, anorexia, weight loss, malaise, pleuritic thoracic pain, tachypnea); increase in sputum purulence; changes in respiratory physical examination; changes in the chest radiographs suggestive of infection; increase of systemic inflammatory markers (C-reactive protein, sedimentation rate); or a positive culture to a microorganism at ≥10^−5^ dilution that has been treated with antibiotics. Data of the number of exacerbations in the year prior to the study were collected from the patients’ medical records.

Anthropometric variables included weight, height, and body mass index [BMI] and were measured with patients barefoot, in underwear, and in standing position, using a standard inextensible measuring tape fixed at the wall (height), and a previously calibrated balance (weight).

For induced sputum analysis, samples were obtained by inhalation of hypertonic saline 4.5% using an ultrasonic nebulizer based on the technique described by Belda [22] and adapted according to the manual of procedures of the Spanish Society of Pneumology and Thoracic Surgery (SEPAR) [23]. The sputum cellularity (%) was determined by May–Grünwald–Giemsa staining and microscopic quantification. The criterion of induced sputum viability was established as < 15% epithelial cells. Sputum cytology included the percentage of neutrophils, eosinophils, lymphocytes, and macrophages (reference values <64.1%, <1.1%, <2.6%, and <86.1%, respectively). Inflammatory biomarkers, including interleukin (IL)-8 (IL-8), tumor necrosis factor alpha (TNF-α), resolvin D1 (RvD1), and polymorphonuclear neutrophil elastase (NE) were measured in sputum samples. As a marker of intestinal inflammation, the calprotectin fecal test was also performed at baseline and at 12 months. 

Forced spirometry was performed using a Sibelmed spirometer (Sibel, S.A.U., Barcelona, Spain) following criteria establish by the American Thoracic Society (ATS) and the European Respiratory Society (ERS) [24]. The following parameters were recorded: forced vital capacity (FVC), FEV_1_, and forced expiratory flow (FEF) between 25% and 75% (FEF_25-75_) expressed in liters (L) and percentage (%) of predicted values for persons of the same sex, age, weight, and height of a reference population according to recommendations of the SEPAR [25] and Roca et al. [26]. Data of spirometries performed in the year prior to the study were collected from the patients’ medical records.

A peripheral blood sample in fasting conditions was drawn at baseline and at visits 4 (6 months) and 6 (end of study) for standard hematological (hemogram) and biochemical parameters (liver and renal function tests) and serum levels of IL-8, TNF-α, and RvD1. Serum and sputum levels of IL-8 and TNF-α were measured using commercially available ELISA kits (RayBio^®^ Human, ref: ELH-IL8-2, biNova Científica, S.L., Barcelona, Spain) for IL-8 and a high sensitive ELISA kit for TNF-α (ref: HEA133Hu-96T, biNova Científica, S.L., Barcelona, Spain). Results were expressed as pg/mL. Sputum and serum concentrations of RvD1 were measured with ELISA using human resolvin D1 plates, 96T format (bioNova Científica, S.L., Barcelona, Spain), and results were expressed as pg/mL. Concentrations of NE in sputum samples were determined using a commercially available kit (PMN Elastase ELISA-DEH 331, Demeditec Diagnostics GmbH, Kiel, Germany) following the manufacturer’s instructions, with results expressed as µg/L. Results of the calprotectin fecal test were expressed as µg/g.

### 2.5. Study Endpoints

The primary endpoint of the study was to compare changes of clinical variables (respiratory function, exacerbation rates, and anthropometric parameters) during the study period between the DHA supplementation and the placebo groups. Secondary endpoints were differences in inflammatory biomarkers at the end of the study between the two study groups.

### 2.6. Statistical Analysis

The sample size was calculated according to data of a longitudinal cohort study in 35 children with CF undergoing sputum induction analysis annually over 3 years, in which a one unit increase in (log) NE corresponded to a 1.1% predicted per year decline in FEV_1_ [27]. Based on a 2% predicted decline in FEV_1_ per year, as an expected worsening of lung function, patients with an increase (log) of at least 1.82 units in NE were considered. Accepting an alpha risk of 5% and a beta risk lower than 20% in a bilateral contrast, 22 participants per group would be needed to detect a 40% difference between the study groups (maximum variability P = Q = 50%).

Frequencies and percentages were used for categorical variables and mean and standard deviation (±SD) or median and interquartile range (IQR) (25th–75th percentile) for continuous variables. The chi-square test was used for the comparison of categorical variables and either the Student’s *t* test or the Mann–Whitney *U* test was used for the comparison of quantitative variables according to conditions of application. The analysis of variance (ANOVA) for repeated measures was used for the within-group comparison of paired variables at 3-month intervals over the study period and in the year prior to the study. Statistical significance was set at *p* < 0.05. SPSS version 25.0 (IBM Corp., Armonk, NY, USA) was used for the analysis of data.

## 3. Results

A total of 22 patients (10 boys and 12 girls) were randomized to the DHA group (*n* = 11) and the placebo group (*n* = 11). In the DHA group, four patients were lost to follow-up; so, seven patients were analyzed in total. In the placebo group, one patient was lost to follow-up and one patient started participation in a CFTR modulator clinical study; so, nine patients were analyzed in total. The mean (SD) age of patients assigned to the placebo group was 11.8 (3.8) years and the mean age of those assigned to the DHA group was 11.4 (2.9). Table 2 shows demographic, anthropometric, and clinical characteristics, and the usual CF medications of the study population. Statistically significant differences between the study groups were not found. Chronic *Pseudomonas aeruginosa* colonization occurred in one patient in the placebo group and in none of the patients in the DHA group. None of the patients had CF-related liver disease or diabetes. None of the patients had gastrointestinal lesions. Pancreatic insufficiency was present in seven (63.6%) patients in the DHA group and in five (45.5%) patients in the placebo group (*p* = 0.668). In relation to medications, none of the patients received long courses of systemic steroids or NSAIDs during the study, except for the sporadic use of ibuprofen as an antipyretic agent.

### 3.1. Pulmonary Function and Exacerbations

The results of the forced spirometry studies are shown in Table 3. Significant differences over the study period in favor of the DHA group were found for FVC (L) and FEV_1_ (L), *p* = 0.004 and *p* = 0.044, respectively. In relation to FEF_25-75_, either expressed as absolute values (liters) or % predicted, there were no significant differences between the study groups during the 12-month study period (Table 2).

**Figure 1:**
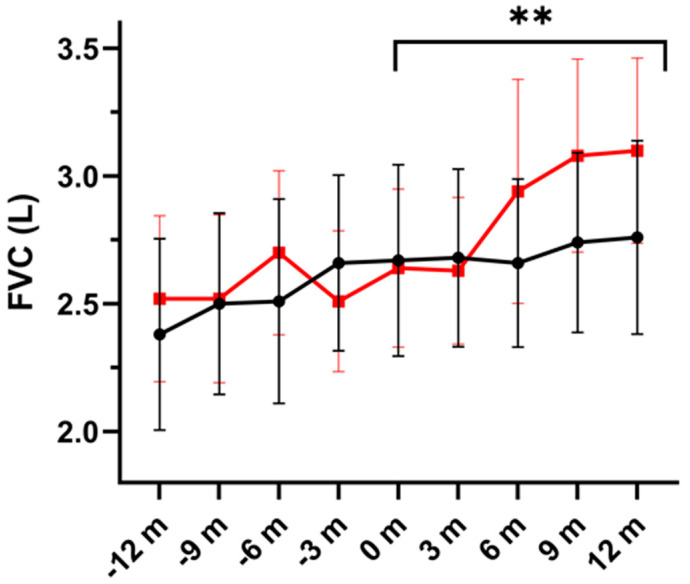
Changes of forced vital capacity (FVC) expressed in liters (mean ± SEM) in the DHA supplementation group (red line) and the placebo group (black line) during the previous year (−12 m to −3 m) and over the study period (0 m to 12 m). Increases in FVC were significantly higher in the DHA group (** *p* = 0.004) (m: months).

**Figure 2:**
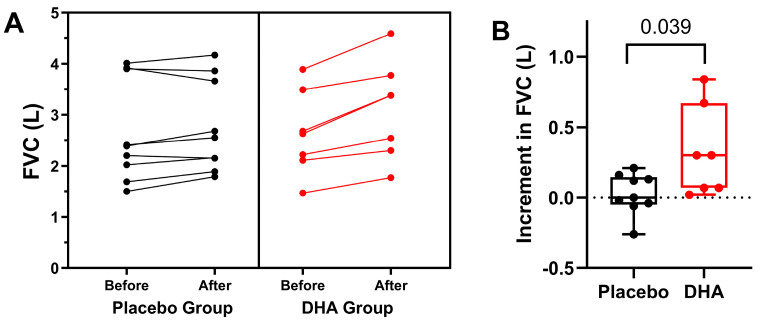
(**A**) Individual values of FVC in the placebo and DHA groups before start of treatment and the end of the observation period. Each dot represents an individual participant. (**B**) Variation in FVC values among study participants. We plotted the variation (12-month vs. baseline) in FVC. Values are presented as the mean ± standard error of mean (SEM), and the box spans from the 25th to the 75th percentiles, with the line representing the mean, and the whiskers indicating the range of minimum and maximum values (*p* = 0.039 using the Mann–Whitney *U* test for the comparison of the two groups).

Figure 1 shows changes in FVC (L) in both study groups during the study period and over the previous year. Individual values in the two study groups during the 12-month study period are depicted in Figure 2.

**Figure 3:**
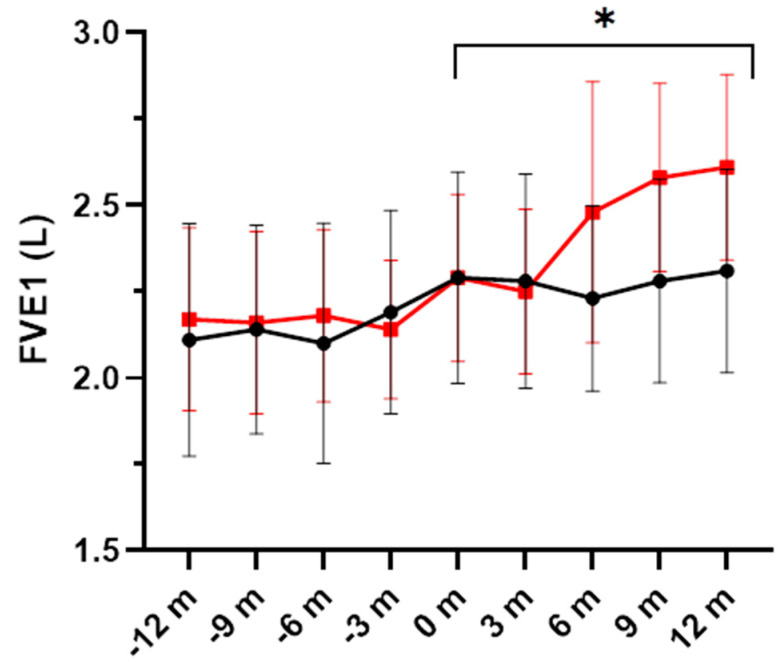
Changes of forced expiratory volume in one second (FEV_1_) expressed in liters (mean ± SEM) in the DHA supplementation group (red line) and the placebo group (black line) during the previous year (−12 m to −3 m) and over the study period (0 m to 12 m). Increases in FEV_1_ were significantly higher in the DHA group (* *p* = 0.044) (m: months).

**Figure 4:**
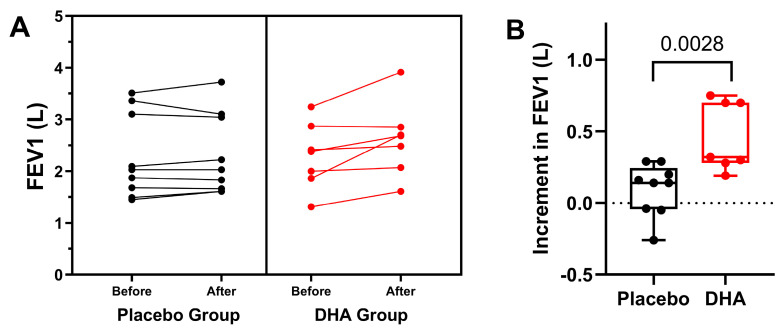
(**A**) Individual values of FEV_1_ in the placebo and DHA groups before start of treatment and at the end of the observation period. Each dot represents an individual participant. (**B**) Variation in FEV_1_ values among study participants. We plotted the variation (12-month vs. baseline) in FVC. Values are presented as the mean ± standard error of mean (SEM), and the box spans from the 25th to the 75th percentiles, with the line representing the mean, and the whiskers indicating the range of minimum and maximum values (*p* = 0.0028 using the Mann–Whitney *U* test for the comparison of the two groups).

In relation to FEV_1_ (L), increases during the study period were significantly higher in the DHA group (*p* = 0.044) (Figure 3). Also, individual values of patients assigned to the DHA or the placebo groups are shown in Figure 4, in which increments in FVE_1_ (L) were higher among patients treated with the DHA supplement (*p* = 0.0028).

The analysis of FVC and FEV_1_, expressed as % predicted, showed a large variability in the two study groups, without statistically significant differences.

The median number of exacerbations in the placebo group was 2 (range 0–2) in the previous year as well as during the study period (*p* = 0.785), whereas in the DHA group, the median number of exacerbations was 2 (range 0–2) in the previous year and 1 (range 0–1) during the study (*p* = 0.492).

### 3.2. Sputum Cellularity 

The results of the sputum cellularity analysis are shown in Table 4. In patients treated with DHA, the percentage of neutrophils remained stable, from a mean of 78.16 ± 6.28% at baseline to 78.13 ± 6.21% at the end of the study. In the placebo group, neutrophils increased from baseline to 6 months, decreasing thereafter. Differences between the study groups were not statistically significant (*p* = 0.385). The percentage of eosinophils showed a decreasing trend in the DHA group, from 4.50 ± 3.81% at baseline to 3.33 ± 2.75 at the end of the study, whereas values were lower but remained stable in the placebo group. Differences in the percentage of eosinophils between the study groups were not significant (*p* = 0.679). The percentage of lymphocytes also showed a large variability throughout the study, with decreasing and increasing trends in the DHA and placebo groups, respectively, but differences were not statistically significant (*p* = 0.342). Changes in the percentage of macrophages increased in the DHA group and decreased in the placebo group, but significant differences were not observed (*p* = 0.328).

### 3.3. Inflammatory Biomarkers

The results of the inflammatory biomarkers analyzed in the induced sputum samples and in serum samples of the two study groups are shown in Table 5. The values of NE in sputum samples increased in both DHA and placebo groups during the study period, but between-group differences were not statistically significant (*p* = 0.675).

The values of RvD1 in the sputum and serum samples were similar in the two study groups after 12 months of supplementation (Table 5). In the sputum samples, the mean levels were 502.24 and 467.34 pg/mL in the placebo and DHA groups, respectively (*p* = 0.298), whereas in the serum samples, the corresponding values were 567.95 and 474.86 pg/mL, respectively (*p* = 0.386).

The analysis of IL-8 showed that the concentration of this cytokine in the sputum samples remained stable during the study period both in patients treated with placebo and those treated with DHA. In the serum samples, the DHA group showed median baseline levels lower than those in the placebo group (4.85 vs. 19.25 pg/mL). However, after 12 months of dietary supplementation, the median values in the DHA group increased from 4.85 to 8.64 pg/mL, but decreased from 19.25 to 9.78 pg/mL in the placebo group (Table 5).

In relation to TNF-α in the sputum samples, median levels remained stable throughout the study period in the two study groups, without significant differences at the end of the study (*p* = 0.699) (Table 5). In the serum samples, median levels of placebo-treated patients remained stable (8.37 to 7.18 pg/mL), but in DHA-treated patients, median values decreased from 8.42 to 6.88 pg/mL. However, statistically significant differences between the study groups were not found.

Fecal calprotectin showed a decrease in both study groups from baseline to the end of the study, but statistically between-group differences were not observed.

### 3.4. Anthropometric Variables

At the end of the study, the mean age of participants was similar (11.42 in the DHA group and 11.72 in the placebo group). As shown in Table 6, there was an increase in weight during the study period in the DHA supplementation group, from a mean of 40.30 ± 14.26 kg at baseline to 44.83 ± 15.25 kg at the end of the study, whereas in the placebo group, weight at baseline was 41.55 ± 14.39 kg and 41.96 ± 11.87 kg at the end of the study. Differences between the study groups were statistically significant (*p* = 0.034).

**Figure 5:**
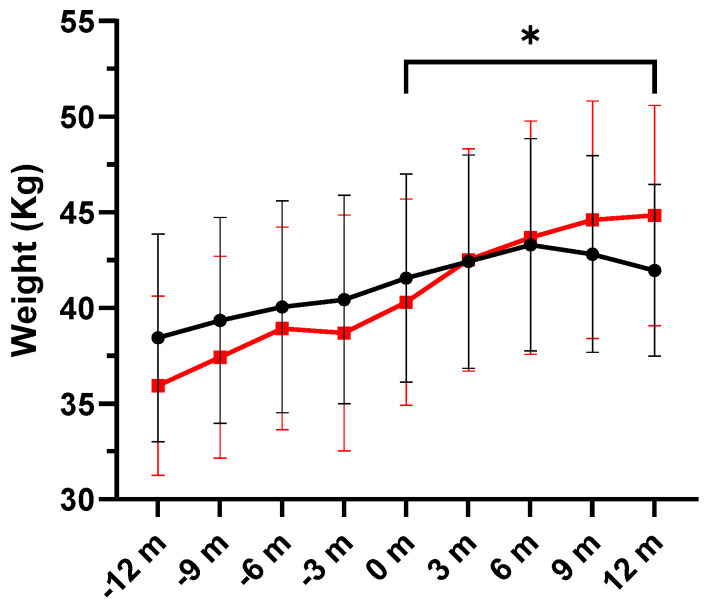
Changes of body weight (mean ± SEM) in the DHA supplementation group (red line) and the placebo group (black line) during the previous year (−12 m to −3 m) and over the study period (0 m to 12 m). Increases in weight were significantly higher in the DHA group (* *p* = 0.034) (m: months).

**Figure 6:**
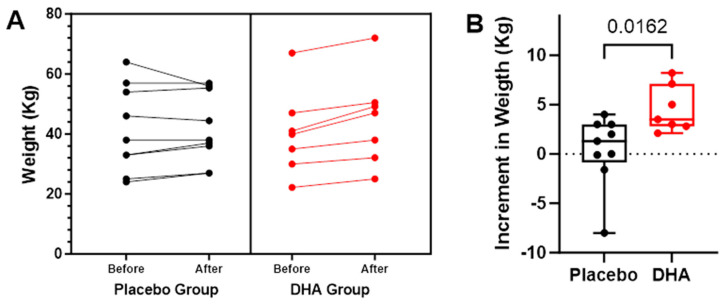
(**A**) Individual values of body weight (kg) in the placebo and DHA groups before start of treatment and at the end of the observation period. Each dot represents an individual participant. (**B**) Variation in body weight (kg) values among study participants. We plotted the variation (12-month vs. baseline), with values presented as the mean ± standard error of mean (SEM), and the box spans from the 25th to the 75th percentiles, with the line representing the mean, and the whiskers indicating the range of minimum and maximum values (*p* = 0.0162 using the Mann–Whitney *U* test for the comparison of the two groups).

Changes in body weight during the study period and over the 12 months prior to the study are shown in Figure 5, with individual variations shown in Figure 6.

**Figure 7:**
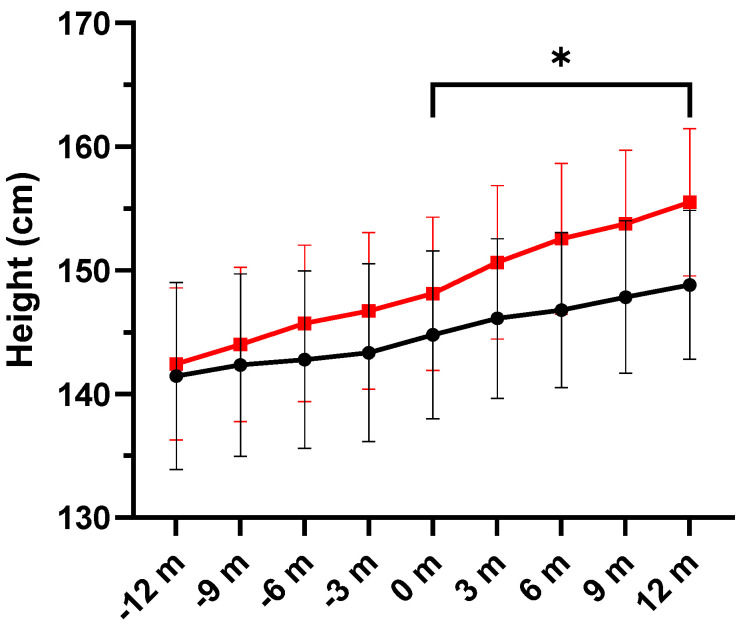
Changes in height (mean ± SEM) in the DHA supplementation group (red line) and the placebo group (black line) during the previous year (−12 m to −3 m) and over the study period (0 m to 12 m). Increases in height were significantly higher in the DHA group (* *p* = 0.029) (m: months).

**Figure 8:**
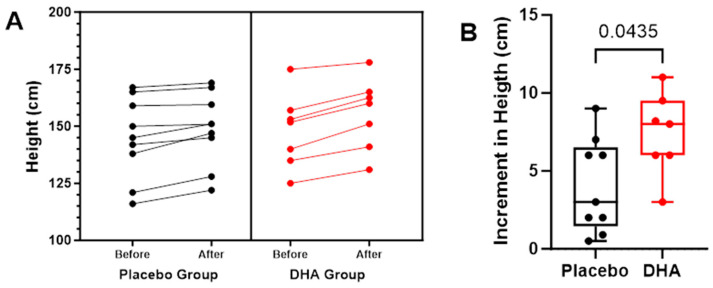
(**A**) Individual values of height (cm) in the placebo and DHA groups before start of treatment and the end of the observation period. Each dot represents an individual participant. (**B**) Variation in height (cm) values among study participants. We plotted the variation (12-month vs. baseline), with values presented as the mean ± standard error of mean (SEM), and the box spans from the 25th to the 75th percentiles, with the line representing the mean, and the whiskers indicating the range of minimum and maximum values (*p* = 0.0435 using the Mann–Whitney *U* test for the comparison of the two groups).

In relation to height, increases were also significantly higher in the DHA group (*p* = 0.026), with differences from baseline to the end of the study of 7.39 and 4.04 cm in the DHA and placebo groups, respectively (Table 6 and Figure 7). Individual variations in height are shown in Figure 8.

Significant changes in BMI were not observed in any of the study groups. 

### 3.5. Adverse Events and Compliance

The study product was safe and well tolerated. One patient treated with DHA reported intermittent abdominal pain without any other accompanied symptom. Although the intake of pancreatic enzymes was increased, abdominal discomfort persisted, and a culture of a stool sample was positive for *Giardia lambia*. Treatment with metronidazole was successful and abdominal symptoms disappeared, but the patient discontinued the study at the parent’s request. A relationship with the study supplement could not be established. Compliance with the dietary supplement was 85.8%, without differences between the study groups.

## 4. Discussion

The present results add data regarding the effect of dietary supplementation with a highly concentrated DHA triglyceride administered for 1 year in a study population of pediatric patients with CF. Randomized controlled trials (RCTs) of omega-3 supplementation in pediatric CF patients are scarce. In a recent systematic review and meta-analysis of the effect of omega-3 supplementation in children and adolescents, only 12 RCTs were selected [28]. Interestingly, the mean age ranged between 8.9 to 18 years and the percentage of males varied between 33.3% and 76.3%. These data are consisted with the demographic characteristics of our population.

The administration of the study product improved pulmonary function, with significant increases in FVC and FVE_1_ expressed in liters as compared with placebo. Other studies using EPA, DHA, or combinations of EPA, DHA, and LA also reported improvements in lung function parameters [11,13,29,30], but differences in the characteristics of the populations (pediatric and adults), doses of omega-3 administered, and duration of supplementation make comparisons difficult. In a recent systematic review and meta-analysis of 12 RCTs of omega-3 supplementation in CF patients younger than 18 years [28], no significant effect on FEV or FVC was found, considering that the heterogeneity was low and insignificant for these two variables. On the other hand, we found a large variability in FEV_1_, FVC, and FEF_25-75_ expressed as % predicted during the year prior to the study, as well as over the 1-year study period.

The occurrence of exacerbations is another important clinical outcome in CF. The median number of exacerbations in our patients was very low, either during the year prior to the study or over the study period (2 episodes in the placebo group and 1 episode in the DHA group), without significant differences. This low number of exacerbations may be explained by the limited sample size and the fact that patients had been regularly followed and had a well-controlled disease. Our results are in agreement with data of other studies, in which differences in the number of exacerbations between patients who received algal DHA administrations for 1 year and patients who received a placebo were not reported [21,31,32]. However, in a study of omega-3 fish oil supplementation, the number of pulmonary exacerbations decreased significantly at 12 months (1.7 vs. 3.0, *p* < 0.01), as did the duration of antibiotic therapy [12].

Cellularity in the induced sputum samples showed a high percentage of neutrophils, representing a very inflamed cell population accumulating in the airways of CF patients [33]. Changes in the differential cell count at the end of supplementation vs. baseline were not found, which coincides with data obtained in a study of adults with CF treated with DHA supplementation at a rate of 3 g daily (Aladin^®^ 500 mg, Laborest, Milan, Italy) for 10 weeks [34]. Moreover, in our study, sputum NE values did not vary significantly in association with DHA supplementation, which is a finding also reported in another RCT of CF patients given a seaweed DHA oil solution or matching placebo for 48 weeks [21].

An important aspect of the study was the assessment of biomarkers of inflammation in sputum and peripheral blood samples. It has been shown that RvD1 levels in CF airways could be a contributing factor to chronic airway inflammation, and that CF patients have a reduced capacity to biosynthesize specialized pro-resolving lipid mediators (SPMs), which contributes to the development and duration of the unwanted inflammation [35,36]. SPMs are multipotent regulators of inflammation and resolution, acting on multiple molecular and cell targets to limit inflammation and return to homeostasis, with emerging results encouraging SPMs as candidate drugs for treating infection and chronic inflammation in CF patients [37]. In the present study, however, the levels of RvD1 in the induced sputum and serum samples did not show significant changes after supplementation with DHA.

The inflammatory process in CF is characterized by the production and release of cytokines and chemokines, among which IL-8 represents one of the most important cytokines [38]. The overexpression of IL-8 and infiltration of neutrophils are the two major markers representing hyperinflammation in CF airways and are associated with the clinical status of CF patients [39]. The median values of serum IL-8 at baseline were lower in the DHA group than in the placebo group (4.85 vs. 19.25 pg/mL), and after supplementation for 1 year, IL-8 levels increased in the DHA group and decreased in placebo-treated patients, although changes between the study groups were not statistically significant. These results are consistent with a lack of changes of inflammatory markers reported by López-Neyra et al. [21] in a RCT of DHA supplementation for 1 year, in which systemic IL-8 was one of the primary variables of the study. In the 1-year RCT of supplementation with algal DHA in pediatric CF patients carried out by Alicandro et al. [31], changes in cytokines were not found either. On the other hand, IL-8 levels in sputum samples showed a decreasing trend in the placebo and DHA group, but differences were not statistically significant, in agreement with data found in the RCT of López-Neyra et al. [21].

Serum and sputum levels of TNF-α showed a decreasing trend in both study groups, without significant differences. Decreases in serum TNF-α levels after supplementation with DHA have been also reported by others [29,31]. In adults with CF, Olveira et al. [11] showed that low-dose supplements of omega-3 and gamma-linolenic fatty acids administered for 1 year was associated with a significant reduction of serum TNF-α and its soluble receptors. In the study of Leggieri et al. [29], CF patients supplemented with DHA showed a decrease in serum levels of IL-8 and TNF-α after 6 months of supplementation, increasing thereafter following 6 months of discontinuation of DHA intake. Although proinflammatory cytokines in sputum samples are useful markers of lung disease activity in CF patients, there is a large variability in TNF-α and IL-8 concentrations in relation to the clinical condition of patients, with peak values on days 3–6 during exacerbation, and a correlation with the colonization of *Pseudomonas aeruginosa* or *Staphylococcus aureus* in the lower airways [40]. Moreover, no significant changes were observed in the levels of RvD1. RvD1 is a lipid mediator that is transiently produced in vivo using DHA as a substrate [41] and is not stable. RvD1 is one of the RvD series (RvD1-RvD6), which are synthesized by leukocytes and endothelial cells during the resolution of inflammation. It has been shown that resolvins stop infiltration by polymorphonuclear leukocytes in vivo, in addition to reducing the expression of proinflammatory mediators [42,43]. Our results cannot provide conclusive evidence regarding changes in inflammatory markers after one year of supplementation with a high-rich DHA triglyceride, which is likely due to the small study population.

On the other hand, changes in anthropometric variables with increases in body weight and height may be expected to occur in children during the growth phase of life, but these increases were greater in the group of DHA-treated participants with statistically significant differences as compared to those in the placebo group. On the contrary, in the analysis of the 12 RCTs included in the systematic review and meta-analysis of Sohouli et al. [28], no significant effect of DHA supplementation on anthropometric parameters was observed.

The present findings should be interpreted while considering the limitations of the study, especially the small sample size. At baseline, patients in both study groups did not show statistically significant differences in age, but the number of pre/post puberty patients in each group was not analyzed. Also, the small sample size and the variability of the cellular profiles and inflammatory biomarkers in the induced sputum samples in relation to the underlying airway inflammation in CF patients may account for dissimilarities between the study groups at baseline. On the other hand, it is difficult to control for inflammatory status or to assess the influence of the pubertal growth spurt. Since participants were not further subdivided into pre- vs. post-puberty groups (e.g., menarche, Tanner stages), the influence of this variable in the present study population remains unexplored. 

This was a clinical trial conducted in daily practice conditions of a rare disease, targeting pediatric patients, and carried out at a single center. A larger study population and an extension of the supplementation period would likely have allowed for more consistent data concerning the clinical relevance of the use of DHA supplementation in pediatric CF patients. The strengths of the study include the design as a RCT and the characteristics of the DHA product, which is a highly concentrated DHA triglyceride that was patented as a cellular antioxidant compound, and which confers a higher effectiveness compared to other *n*-3 PUFA products that were analyzed in previous systematic reviews [14,15,28].

## 5. Conclusions

In the present RCT, the use of a high-rich DHA triglyceride supplement (Tridocosahexaenoin-AOX^®^ 70%) at a dose of 50 mg/kg/day administered for 1 year in patients with CF, with a mean age of 11.7 years, was associated with significant improvements of pulmonary function parameters (FVC and FVE_1_ expressed in liters) and a decrease in the number of exacerbations as compared with placebo. Significant changes in inflammatory biomarkers, either in induced sputum or peripheral blood samples, were not found. Significant increases in weight and height were also observed among DHA-supplemented patients. The administration of the study product was safe and well tolerated. Further RCTs with larger study populations and the use of higher quality DHA supplements for prolonged periods are necessary to confirm the present findings.

## Figures and Tables

**Table 1:** Doses and daily intakes of DHA.

Weight, kg	Dose, mg	Number of Capsules	Daily Intakes *
≥13 to 17	700	2	One or two
≥18 to 24	1050	3	One or two
≥25 to 30	1400	4	Two (2 capsules–2 capsules)
≥31 to 36	1750	5	Two (2 capsules–3 capsules)
≥37 to 43	2100	6	Two (3 capsules–3 capsules)
≥44 to 49	2450	7	Three (3 capsules–1 capsule–3 capsules)
≥50	2800	8	Three (3 capsules–2 capsule–3 capsules)

* At the time of breakfast, lunch, and/or dinner according to the number of daily doses.

**Table 2:** Demographic, anthropometric, and clinical characteristics, and usual medication of the study population.

Variables	Patients with Cystic Fibrosis (*n* = 22)		
	DHA Group (*n* = 11)	Placebo Group (*n* = 11)	*p* Value
Gender, male, *n* (%)	7 (63.6)	3 (23.3)	0.160
Age, years, mean (SD)	10.9 (3.2)	12.5 (3.8)	0.381
Weight, kg, mean (SD)	39.7 (17.4)	43.4 (14.6)	0.803
Height, cm, mean (SD)	145.2 (20.2)	149.3 (19.0)	0.743
Body mass index, kg/m^2^, mean (SD)	17.9 (2.9)	19.3 (2.4)	0.501
Mutations, *n* (%) *			
Severe/severe	7 (63.6)	5 (45.5)	0.668
Severe/mild	3 (27.3)	4 (36.4)	1.0
Mild/mild	1 (9.1)	2 (18.2)	1.0
Genotype, *n* (%)			
ΔF508/ΔF508	3 (27.3)	1 (9.1)	0.580
ΔF508del/other	5 (45.5)	7 (63.6)	0.668
Other/other	3 (27.3)	3 (27.3)	1.0
Pancreatic insufficiency, *n* (%)	7 (63.6)	5 (45.5)	0.668
Chronic *S. aureus* infection, *n* (%)	3 (27.3)	2 (18.2)	1.0
Chronic *P. aeruginosa* infection, *n* (%)	0	1 (9.1)	1.0
FEV_1_, %, mean (SD)	92.36 (9.8)	97.39 (15.5)	0.373
Diagnosis by neonatal screening, n (%)	11 (100)	10 (90.9)	>0.999
Exercise (≥ 3 times/week), n (%)	8 (72.7)	6 (54.5)	0.659
CF-related diabetes, n (%)	0	0	1.0
CF-related liver disease, n (%)	0	0	1.0
Allergic bronchopulmonary aspergillosis (ABPA), n (%)	0	0	1.0
Usual medication, n (%)			
Nebulized hypertonic saline	7 (63.6)	9 (81.8)	0.635
Nebulized dornase alfa	4 (36.4)	8 (72.7)	0.198
Nebulized sodium bicarbonate	5 (45.5)	4 (36.4)	>0.999
Azithromycin 3 times a week	1 (9.1)	2 (18.2)	>0.999
Inhaled corticosteroids	4 (36.4)	6 (54.5)	0.670
Multivitamins	11 (100)	11 (100)	1.0
Pancreatic enzymes	7 (63.6)	5 (45.5)	0.688

* Severe mutations: mutations with mild or minimal function of *CFTR* (group I, II or III mutations); mild mutations: mutations with residua *CFTR* function (group IV, V, VI or VII muta-tion); SD: standard deviation.

**Table 3:** Changes of pulmonary function parameters during the 12-month study period.

Variables	Baseline	3 Months	6 Months	9 Months	12 Months	*p* Value
FVC, L,						
Placebo	2.67 ± 0.99	2.60 ± 0.92	2.66 ± 0.87	2.74 ± 0.93	2.76 ± 1.0	0.004
DHA	2.64 ± 0.82	2.63 ± 0.76	2.94 ± 1.16	3.08 ± 1.0	3.10 ± 0.96	
FVC, %						
Placebo	98.55 ± 14.69	97.88 ± 11.01	96.44 ± 8.54	96.01 ± 31.86	96.84 ± 14.69	0.317
DHA	91.71 ± 10.09	87.99 ± 11.96	86.55 ± 5.90	93.81 ± 6.36	94.38 ± 7.44	
FEV_1_, L						
Placebo	2.29 ± 0.81	2.28 ± 0.82	2.23 ± 0.71	2.28 ± 0.78	2.31 ± 0.78	0.044
DHA	2.29 ± 0.64	2.25 ± 0.63	2.48 ± 1.00	2.58 ± 0.72	2.61 ± 0.71	
FEV_1_, %						
Placebo	98.55 ± 16.92	95.44 ± 12.63	95.61 ± 10.86	93.96 ± 6.32	94.63 ± 19.96	0.429
DHA	93.28 ± 11.57	87.55 ± 7.58	89.68 ± 6.46	93.96 ± 6.32	93.44 ± 8.74	
FEF_25-75_, L						
Placebo	2.50 ± 0.62	2.42 ± 0.84	3.07 ± 1.15	2.45 ± 1.13	2.81 ± 1.22	0.476
DHA	2.66 ± 0.61	2.78 ± 0.80	3.26 ± 0.94	3.23 ± 0.77	3.68 ± 2.06	
FEF_25-75_, %						
Placebo	93.33 ± 22.86	86.11 ± 22.84	93.33 ± 18.26	82.04 ± 25.83	85.75 ± 20.53	0.493
DHA	92.71 ± 15.53	85.45 ± 12.43	91.35 ± 9.70	92.04 ± 12.08	87.69 ± 17.22	

FVC: forced vital capacity; FEV_1_: forced expiratory volume in one second; FEF_25-75_: forced expiratory flow between 25% and 75%. DHA: docosahexaenoic acid. Data as mean ± standard deviation.

**Table 4:** Changes in induced sputum cellularity in the two study groups.

	Placebo Group			DHA Group			*p* Value
	Baseline	6 Months	12 Months	Baseline	6 Months	12 Months	
Neutrophils, %	56.70 ± 24.20	72.12 ± 13.09	61.62 ± 19.09	78.16 ± 6.28	74.76 ± 18.90	78.13 ± 6.21	0.385
Eosinophils, %	1.62 ± 0.19	1.01 ± 0.55	1.65 ± 2.10	4.50 ± 3.81	2.76 ± 1.51	3.33 ± 2.75	0.679
Lymphocytes, %	6.90 ± 4.03	6.93 ± 5.94	24.35 ± 31.45	5.53 ± 3.78	6.73 ± 1.11	3.27 ± 0.86	0.342
Macrophages, %	27.75 ± 24.47	15.52 ± 13.25	23.20 ± 24.24	3.86 ± 1.79	13.63 ± 8.86	8.97 ± 28.26	0.328

DHA: docosahexaenoic acid. Data expressed as mean ± standard deviation.

**Table 5:** Changes in inflammatory biomarkers at the end of the study as compared with baseline in the two study groups.

Variables	Placebo Group		DHA Group		*p* Value
	Baseline	12 Months	Baseline	12 Months	
Sputum samples					
NE, µg/L	49.38 ± 53.21	79.50 ± 125.82	16.80 ± 10.42	49.60 ± 61.67	0.675
RvD1, pg/mL	521.29 ± 92.52	502.24 ± 73.49	481.87 ± 63.14	467.34 ± 60.43	0.298
IL-8, pg/mL *	9310.8 (6285.2–14,825.6)	8285.9 (5796.4–8861.2)	6449.4 (1719.4–21,146)	5756.6 (5556.7–7535.8)	0.248
TNF-α, pg/mL *	4.03 (3.56–5.21)	3.69 (3.28–3.85)	4.09 (3.91–7.45)	3.71 (3.34–4.68)	0.699
Serum samples					
RvD1, pg/mL	556.80 ± 157.91	567.95 ± 147.80	483.30 ± 157.91	474.86 ± 127.91	0.386
IL-8, pg/mL *	19.25 (5.47–45.01)	9.78 (3.97–44.84)	4.85 (3.60–6.40)	8.64 (3.89–38.19)	0.068
TNF-α, pg/mL *	8.37 (7.33–9.47)	7.18 (6.34–7.62)	8.42 (7.57–9.24)	6.88 (6.14–7.38)	1.0
Fecal samples					
Fecal calprotectin, µg/g	66.22 ± 65.94	41.78 ± 37.36	140.92 ± 102.79	96.40 ± 87.69	0.293

DHA: docosahexaenoic acid; NE: neutrophil elastase; RvD1: resolvin D1; IL-8: interleukin 8; TNF-α: tumor necrosis factor alpha. Data as mean ± standard deviation or * median and interquartile range in parenthesis.

**Table 6:** Changes in anthropometric variables at the end of the study as compared with baseline in the two study groups.

Variables	Study Period					*p* Value
	Baseline	3 Months	6 Months	9 Months	12 Months	
Weight, kg						
Placebo	41.55 ± 14.39	42.42 ± 14.76	43.30 ± 14.69	42.81 ± 13.60	41.96 ± 11.87	0.034
DHA	40.30 ± 14.26	42.51 ± 15.36	43.67 ± 16.16	44.61 ± 16.42	44.83 ± 15.25	
Height, cm						
Placebo	144.78 ± 17.95	146.10 ± 17.06	146.79 ± 16.58	147.83 ± 16.32	148.82 ± 15.93	0.026
DHA	148.11 ± 16.38	150.64 ± 16.42	152.54 ± 16.16	153.78 ± 15.68	155.50 ± 15.79	

Data expressed as mean ± standard deviation; DHA: docosahexaenoic acid.

## Data Availability

Study data are available from the first author (R.A.-V.) upon request. The data are not publicly available due to privacy and ethical restrictions.
